# Self-Perceived Stress in Association with Emotional Experiences Following Patient Death and Coping Adequacy among Clinical Nurses in Lithuania: A Cross-Sectional Study

**DOI:** 10.3390/jcm13092533

**Published:** 2024-04-25

**Authors:** Marius Baranauskas, Marius Kalpokas, Ingrida Kupčiūnaitė, Jurgita Lieponienė, Rimantas Stukas

**Affiliations:** 1 Faculty of Biomedical Sciences, State Higher Education Institution Panevėžys College, 35200 Panevėžys, Lithuania; marius.kalpokas@panko.lt (M.K.); ingrida.kupciunaite@panko.lt (I.K.); jurgita.lieponiene@panko.lt (J.L.); 2 Department of Public Health, Institute of Health Sciences, Faculty of Medicine, Vilnius University, 01513 Vilnius, Lithuania; rimantas.stukas@mf.vu.lt

**Keywords:** coping strategies, chronic stress, emotions, healthcare, mental health, nurses, patient death, public health

## Abstract

(1) **Background**: Stress is defined as a psychological sensation related to a change in both human physiology and behavior in response to a threat or challenge. As the prevalence of stress is increasing globally, nurses represent a risk group for exposure to stress-related psychological alterations. The aim of this study was to explore how clinical nurses in Lithuania cope with the perceived chronic stress in relation to additional emotional experiences following the death of a patient. (2) **Methods**: During a four-week period of October 2023, a total of 283 female nurses, aged between 20 and 70 years old, were enrolled in a single cross-sectional study. The PSS-10 (perceived stress scale) and the Brief-COPE (Coping Orientation to Problems Experienced) questionnaires were applied to assess the level of self-perceived stress and the use of coping styles along with the adequate strategies. Furthermore, the nurses’ emotions, evoked following patient death, were classified depending on their valence. Both the multiple linear and logistic regression statistical analyses were conducted to examine the associations between dependent and independent variables. (3) **Results**: The study identified that more than half of the nurses experienced patient death several times a month. A moderate-to-high level of the symptoms of mental chronic stress were suffered by 76% of caregivers. The psychological arousal following the death of a patient may induce the occurrence of emotional complexity that refers to competitive feelings, namely, helplessness (adjusted odd ratio (AOR) 1.7, 95% confidence interval (95% CI) 1.1; 2.9), disappointment (AOR 1.9, 95% CI 1.1; 3.5), anxiety (AOR 1.9, 95% CI 1.2; 4.2), and guilt (OR_adj_ 4.7, 95% CI 1.4; 5.7), and can serve as a potential trigger for chronic stress development in clinical nurses. In terms of emotion-focused stress control (β 0.1, 95% CI −0.1; −0.2, *R*^2^ = 0.12), Lithuanian nurses had low control of the perceived chronic stress. The use of problem-focused coping (β −0.2, 95% CI −0.3; −0.1, *R*^2^ = 0.09) was also not addressed to an appropriate extent. The use of the avoidance coping style was associated with moderate–high levels of chronic stress perceived by nurses (β 0.5, 95% CI 0.4; 0.7, *R*^2^ = 0.24). Furthermore, the avoidant coping strategy related to behavioral disengagement was significantly related to resilience to chronic stress in an adverse way. The cognitive process of self-blame was considered as a meaningful maladaptive behavior component for magnifying chronic stress in nurses. (4) **Conclusions**: The study highlights the need to implement the recommendations for boosting the nurses’ mental health. Hospitals should contribute to psychological assistance along with providing necessary training on stress-coping strategies for clinical nurses. In order to promote the sustainability of mental health through additional social support interventions, it would be useful to upgrade the clinical nurses’ daily routines with mindfulness-based trainings.

## 1. Introduction

Stress is defined as a psychological sensation related to a change in both human physiology and behavior in response to a threat or challenge. Although acute stress is considered to be a certain component for basic survival, at the same time, a wide array of health problems can be evoked by chronic stress [1]. According to The Diagnostic and Statistical Manual of Mental Disorders (DSM-5) [2], the disorders triggered by specific stressful and traumatic experiences are classified as acute stress disorders, which, in turn, are categorized into trauma- and stressor-related disorders. The perception of chronic stress is delineated as the mental response to stress [3]. As one of the most extensively used instruments, the perceived stress scale (PSS) is applied to assess the levels of the perceived chronic stress in a variety of types of population groups [4]. PSS is usually utilized to measure the levels of perceived stress ascribed to individuals’ lives instead of centering on a fixed event or stressor [5]. Taking into account statistics, in 2022, moderate to severe symptoms of perceived stress worldwide accounted for 66% and 57% of adults aged between 18 and 35, respectively [6]. The prevalence of stress is increasing globally, including the members of healthcare staff, especially nurses. For example, the meta-analysis conducted during the pandemic period of coronavirus disease (COVID-19) found that the prevalence estimate for stress overload among nurses from 40 countries ranged between 10% and 84% with an average value of 43% [7]. These scientific data were consistent with the policy brief by The World Health Organization (WHO), alluding to a large increase in the global prevalence of mental issues within the period of the COVID-19 pandemic [8]. In this global context, clinical nurses represented a risk group for exposure to stress [9] and induced psychological alterations [10].

The main stressors encountered by persons in the nursing profession can be categorized by two factors related to the characteristics specific to this profession and associated with the organizational structure of labor [11]. The category of work-related stress agents comprises a considerable amount of work [12], night-shift nursing [13], conditions of employment, hierarchy, and a strong sense of responsibility. Meanwhile, the specificity of the nursing profession is covered by a continuous proximity to both adversity and the death of patients [11]. Chronic mental stress often affects the health of nurses leading to occupational stress and burnout [14,15], sleep disorders [16], the occurrence of both anxiety and depressive symptoms [17], errors in nursing and decreased patient satisfaction [18], negative outcomes of patient care, and sometimes even reduced financial well-being of healthcare organizations [19]. The acute stress in caregivers caused by loss of a patient can lead to emotional conditions associated with negative feelings and, as a consequence, outcomes such as higher levels of chronic stress related to palliative care [20], symptoms of mild death anxiety [21], and personal as well as professional transformations [22]. Acute stress does not usually serve as a health risk developer [23]. However, if a nurse loses their ability to adapt to changing conditions, there are concerns for the occurrence of an imbalance, which can become pathological because psychological resistance is not considered constant throughout an individual’s life and can fluctuate from day to day. Furthermore, the accumulation of negative emotions such as helplessness, sadness, grief, anxiety, frustration, and even guilt [24,25] following the death of a patient may result in chronic stress, which substantially leads to the emerging state of exhaustion [26]. Therefore, our study considered and included emotional states (in terms of the expression of emotional quality and complexity [23]) of nurses after the death of a patient as potential risk factors for the persistence of chronic stress perceived by nurses.

Cognitive and behavioral efforts oriented towards various coping styles and strategies applied by a person are among the most important factors that determine the effectiveness of chronic stress management that could lead to the potential development of emotional exhaustion, depersonalization, reduced personal accomplishment, or the manifestations of fatigue [25,27]. Hence, coping styles trigger a response to threats and/or challenges in order to prevent or at least reduce negative stress-induced outcomes [28]. It should be highlighted that both unconsciously and consciously, as a means to deal with chronic stress, individuals use a diversity of coping strategies that may be assessed via self-report questionnaires [29]. Whilst some research used a two-dimensional (in terms of active and passive/avoidant coping) classification of coping strategies, our study was based on the Theory of Stress and Coping established by Lazarus and Folkman [27], referring to two processes, namely, ‘cognitive appraisal and coping’. Considering that nurses have stronger emotional responses and are more willing to apply a problem-focused coping style [30,31] with a better psychological outcome [32,33], our study focused on more active adaptive coping strategies attributed to both problem-focused and emotion-focused coping styles [34]. Meanwhile, avoidant coping was defined as maladaptive, assuming that passive strategies were used by individuals forced to contend with stressful events [35]. Even though the potential health effects of the avoidance style are less researched, some scientists have reported an association between the use of maladaptive coping strategies and the inferior mental health outcomes of nurses [36]; Dougall et al. [37] and Tripathy et al. [38] have found a relationship between a passive approach to coping and poorer immune responses. Also, other scientists have revealed poorer values of life quality in relation to the application of an avoidant coping style in patients [39,40,41].

The scientific literature reflects that both perceived chronic stress and coping strategies are of interest to researchers. Nevertheless, there is an existing gap in the research on the perceived stress of nurse cohorts after the COVID-19 pandemic; there is also a paucity of data from Lithuania. In addition, according to WHO data, the number of people aged 80 and over is expected to triple to 426 million between 2020 and 2050 [42]. If life expectancy and the ageing population increase, the trends for the need of palliative care along with the number of patient deaths in treatment facilities will also increase. Evidently, nurses are exposed to various stressful factors and experience emotional vulnerability when providing end-of-life care to patients [24]. Although caregivers have a risk of exposure to various negative emotions related to the death of a patient, we hypothesized that negative emotional statuses resulting from acute stressful situations potentially transform into perceived chronic stress in nurses. On the other hand, we assumed that, in the case of stress events leading to chronic stress perceived by nurses, the use of coping styles may not be effective due to the narrow range of interventions included in preventive programs [43], which promote the mental health sustainability of healthcare workers in Lithuania and are targeted solely towards reducing psychological violence in the workplace and preventing excessive alcohol consumption. Thus, this study aimed to explore potential risk factors of interest for chronic stress development in a sample of clinical nurses. To meet this aim, the following research questions (RQ) were formulated:

**RQ1:** Do emotional experiences after dealing with patient death have a relation to nurses’ self-perceived stress?

**RQ2:** Does the use of coping strategies have an association with the self-reported perception of nurses’ stress?

## 2. Materials and Methods

### 2.1. Study Design, Participants, and Data Collection

During a four-week period in October 2023, an online-based cross-sectional study was conducted in the largest cities (Vilnius, Kaunas, and Panevėžys) of Lithuania.

Per one thousand residents of the Republic of Lithuania there are 7.7 nurses. With a total population of 2,708,632 inhabitants in Lithuania, a target population of more than 20,000 employed nurses was calculated (N = 20,856). A priori representative sample size (n = 264) with a confidence level of 95% and a marginal error of 6% was calculated from the group of employed nurses using OpenEpi version 3.01 [44].

The simple random sampling technique was employed during the recruitment procedure of nurses. The study participants were recruited through the websites of official social media groups for nurses, namely, ‘Lithuanian nurses’ (n = 12,000), ‘Nurses’ (n = 2300), and ‘Nursing’ (n = 1899), administrated in Lithuania. All the nurses from the general set were given equal access to enter the study sample. The nurses who accessed the target sites via a link provided by the social media admins could complete the survey. A total of 20,856 nurses were approached to participate in this observational study. After the study participants had given consent and were involved in the survey, they were requested to navigate to the website and fill in an online questionnaire. As the built-in questionnaire was integrated in an online survey management system, a web-based E-survey research application (Apklausa version 204) was applied to collect information from study participants (https://apklausa.lt) (accessed on 26 March 2024).

**Figure 1:**
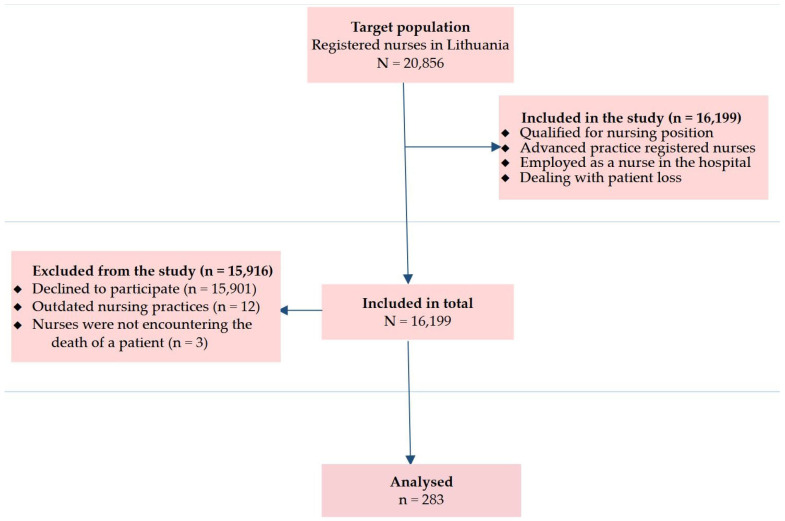
Flowchart of nurse inclusion and exclusion criteria.

The participant inclusion criteria were as follows: (a) persons qualified for nursing position; (b) advanced practice registered nurses; (c) persons employed as nurses in hospitals of major cities and districts of Lithuania; and (d) nurses dealing directly and personally with patient loss in the workplace. During the survey execution, out of the eligible population of 16,199 nurses, 15,916 participants were excluded from the study depending on a shortage of inclusion criteria or failing to complete the questionnaires. The exclusion criteria were defined as follows: (a) nurses who abstained from participating in the study (n = 15,901); (b) study participants with outdated nursing practices (n = 12); and (c) nurses who had not encountered the death of a patient (n = 3). As a consequence, the data of female nurses (n = 283) aged 20 to 70 within February–March 2024 were included and analyzed. A more in-depth analysis of the study recruitment process is represented in Figure 1. 

### 2.2. Measures

An online anonymous self-reported questionnaire for nurses consisted of three parts. The first part of the questionnaire was constructed by the study authors with a focus on the subjects’ demographic and occupational characteristics such as age (in years), work experience (in years), educational attainment (with the response options ‘University’ or ‘College’), marital status (with the response options ‘in a relationship’, ‘divorced’, ‘married’, ‘widowed’ or ‘single’), workplace (with response alternatives of ‘an intensive care unit’, ‘a surgical profile unit’, ‘a therapeutic profile unit’, or ‘an emergency profile unit’), nursing shifts (with the response choices ‘day shifts’, ‘night shifts’, or ‘mixture of day and night shifts’), and experiences with patient loss (with the response options ‘never’, ‘one time a year’, ‘several times a year’ or ‘several times a month’, while each answer was scored from 1 to 4). Additionally, the first part of the questionnaire was supplemented by the questions concerning the nurses’ emotions and feelings triggered by the death of a patient in the workplace. In line with the recommendations found in the scientific literature [24], the issues about the feelings and emotions after dealing with the death of a patient have also been investigated by the authors of this study. The questions referring to the nurses’ responses on raised feelings and emotions such as guilt, compassion, indifference, disappointment, sadness, depressive mood, despair, calmness, anger, helplessness, grief, and anxiety after death of a patient were measured on a nominal scale (with the response choices ‘Yes’ or ‘No’). The comparison of average results obtained from individual answers to the questions about the emotional state of nurses made it possible to determine both the dominant emotions toward death and methods for dealing with complex emotions (in terms of the quantity of emotions) following the patient loss. The nurses’ emotions, which were evoked following patient death, were categorized based on their valence [45].

In the second and third sections of the survey, the PSS-10 (perceived stress scale) [5] and the Brief-COPE (Coping Orientation to Problems Experienced) questionnaires were used to assess the level of self-perceived stress and the use of coping styles along with strategies. Further details on the instruments used in the study are shown in Table 1.

#### 2.2.1. Perceived Stress Scale (PSS-10)

The PSS-10 is a popular instrument for assessing perceived chronic stress [46]. The PSS-10 consists of 10 items relating to feelings and thoughts associated with personal problems and behaviors that a participant has experienced in the last month. The answer to each item on the PSS-10 was scored between 0 (‘never’) and 4 (‘very often’) according to a five-point Likert scale. The cut-off scores of 13 and 26 were used to arrange the total PSS-10 score into low (≤13 points), moderate (14–26 points), and high levels (27–40 points) of the perceived chronic stress [47]. 

**Table 1:** Characteristics of instruments used in the cross-sectional study.

Instrument	Content	Scaling
Demographic and occupational questionnaire (7 items). Questions concerning the emotions and feelings in nurses triggered by the death of a patient (12 items) [24].	Age, work experience, educational attainment, marital status, workplace, nursing shifts, and experiences with patient loss. Issues about the feelings and emotions after dealing with the death of a patient: guilt, compassion, indifference, disappointment, sadness, depressive mood, despair, calmness, anger, helplessness, grief, and anxiety.	Ratio scales. Ordinal scale: 1 = ‘never’ to 4 = ‘several times a month’. Nominal scale: 0 = ‘No’ to 1 = ‘Yes’.
Perceived Stress Scale (PSS-10) (10 items) [4,5,48].	PSS-10 helps to assess how overloaded, unpredictable, and uncontrolled persons perceive their lives. PSS-10 questions relate to feelings and thoughts that a person has experienced in the last month.	Five-point Likert scale: 0 = ‘never’ to 4 = ‘very often’; high score = stressful situation was experienced more often. The cut-off scores of 13 and 26 are used to arrange the total PSS-10 score into the low, moderate, and high levels of the perceived chronic stress [47].
Coping orientation to problems experienced inventory (Brief-COPE) (28 items) [34].	The Brief-COPE was constructed to measure the effective and ineffective ways to cope with a stressful life event. This scale is often used in healthcare settings to assess the patients’ emotional response to difficult circumstances. In general, Brief-COPE consists of 3 dimensions with 14 sub-dimensions and assesses 3 styles of coping: (1) problem-focused coping, (2) emotion-focused coping, and (3) avoidant coping [49].	Four-point Likert scale: 1 = ‘I haven’t been doing this at all’ to 4 = ‘I’ve been doing this a lot’. Scores are presented for three overarching coping styles as average scores, delineating the degree to which the person was engaged in a coping style. There is no cut-off score for the Brief-COPE scale and its subscales.

According to Cohen et al., the internal reliability of the original version of PSS-10 was high when Cronbach’s alpha fluctuated between 0.84 and 0.86 [4]. In the Lithuanian version, the Cronbach’s alpha of PSS-10 was 0.82 [48].

#### 2.2.2. Coping Strategies Inventory (Brief-COPE)

This method is often used to evaluate dispositional coping, i.e., the assessment of resilience to perceived chronic stress. The Brief-COPE tool includes 28 items that form 3 dimensions and 14 sub-dimensions (2 items in each sub-dimension) of styles and strategies to cope with chronic stress [34,49]. The Brief-COPE assesses three styles of coping: (1) problem-focused coping, (2) emotion-focused coping, and (3) avoidant coping. The dimensions of the 3 Brief-COPE scales include 14 facets as follows: ‘active coping’ (items 2; 7), ‘positive reframing’ (items 12; 17), ‘informational support’ (items 10; 23), ‘planning’ (items 14; 25), ‘positive reframing’ (items 12; 17), ‘emotional support’ (items 5; 15), ‘venting’ (items 9; 21), ‘self-blame’ (items 13; 26), ‘acceptance’ (items 20; 24), ‘self-distraction’ (items 1; 19), ‘religion’ (items 22; 27), ‘humor’ (items 18; 28), ‘behavioral disengagement’ (items 6; 16), ‘denial’ (items 3; 8), and ‘substance use’ (items 4; 11). The answer to each statement on the Brief-COPE was scored between 1 (‘I haven’t been doing this at all’) and 4 (‘I’ve been doing this a lot’), according to a four-point Likert scale. Although cut-off scores were not used to categorize the total Brief-COPE score, the scores were presented as average values, delineating the degree to which the study participants were engaged in different coping styles. As the Brief-COPE was constructed to measure the efficient and non-efficient ways to cope with stressful life events, a Lithuanian translation [48,50] of the Brief-COPE questionnaire was developed. The Cronbach’s alpha values for the Brief-COPE subscales related to problem-focused coping, emotion-focused coping, and avoidant coping were 0.76, 0.67, and 0.79, respectively. The total Brief-COPE score had a Cronbach’s alpha of 0.82 [48].

### 2.3. The Statistical Data Analysis

This single cross-sectional study was performed in agreement with the checklist of Strengthening the Reporting of Observational Studies in Epidemiology (STROBE) [51].

The statistical data analysis was performed using the Statistical Package for the Social Sciences (IBM SPSS Statistics) version 25.0 for Windows (IBM Corp, Armonk, NY, USA). The statistical visualization of study data was performed using SPSS software version 25.0 along with the free and open source software LibreOffice version 7.6.4.

The normality of data was tested using the Kolmogorov–Smirnov test. All categorical data were represented using the relative frequency tables. The differences and correlations in categorical variables (age categories, work experience, educational attainment, marital status, workplace, nursing shifts, and the expression of complex emotions after patients’ death) between groups of the nurses with different levels of the perceived chronic stress were assessed using Cramer’s V (*V*) and phi (*φ*) correlation coefficients. The values of *V* and *φ* were interpreted as follows: 0 ≤ |*V or φ*| < 0.2 (‘weak correlation’), 0.2 ≤ |*V or φ*| < 0.4 (‘moderate correlation’), and |*V or φ*| ≥ 0.4 (‘relatively strong and strong correlation’). 

The measures of central tendency (mean (M) (standard deviation (SD)) were applied to disclose the gross scores of the data under analysis. The paired *t*-test was used to assess the differences between the mean scores of coping strategies for chronic stress.

The multiple linear regression models were obtained to assess the association between perceived chronic stress as a dependent variable and independent variables, namely, the expression of emotional complexity and different dimensions along with sub-dimensions of the Brief-COPE scale. In the first linear regression model, the confounding variable was nursing experience. The remaining models of linear regression had covariates related to nursing experience, dealing with the frequency of patient loss and the expression of complex emotions following the death of a patient. The coefficient of determination (*R*-Squared (*R*²)) was calculated to assess the goodness-of-fit of each linear regression model.

The logistic regression analyses were performed to calculate the adjusted odds ratios (AORs) and 95% confidence intervals (CIs) as well as to disclose whether an association persists between the perceived chronic stress of nurses (a dependent variable) and the emotions experienced after the death of a patient (independent variables). The dependent variable PSS-10 was converted to the dichotomous form (1—PSS-10 score ≤ 13 points (reference category); 2—PSS-10 score: from 14 to 40 points). The logistic regression models were adjusted for nursing experience.

The critical value of the significance level was set as α = 0.05 in all statistical tests performed.

## 3. Results

### 3.1. The Descriptive and Frequency Analyses

The sample under analysis included female nurses (n = 283) with a mean age of 38.7 years who declared nursing experience between 0.5 and 50 years (M = 13.2, SD = 11.6 years). The vast majority of study participants (60.1%) experienced patient loss several times a month. Most of the nurses were married (51.9%) and had university degrees (53%). All the nurses working in healthcare institutions were registered and collaborated with healthcare providers in large cities of Lithuania, namely, Vilnius, Kaunas, and Panevėžys. Based on the collected data, it was displayed that the nurses worked at four hospital departments: an emergency profile unit (43.8%), a therapeutic profile unit (24.0%), an intensive care unit (23.3%), and a surgical profile unit (8.8%). Taking into account the nature of the nursing shifts, 22%, 14.5% and 52.9% of the study participants were engaged as day-time, night-time, and mixed-shift workers, respectively.

The PSS-10 questionnaire allowed us to assess the magnitude of chronic stress that was subjectively perceived. As shown in Table 2, 68.6% of the nurses experienced a moderate level of perceived stress and 7.8% of the participants reported a high level of chronic stress (M (SD) = 17.8 (5.7)).

Table 3 shows the distribution of study participants (in percentage) by the level of chronic stress the nurses perceived in the last month according to the demographic and occupational characteristics and their experiences with patient loss. No statistically significant correlation was found between the higher level of perceived stress and the age categories, educational attainment, marital status, workplace, and nursing shifts (*p* > 0.05). On the contrary, in agreement with the cut-off of 13 on the PSS-10, more than 80% of the nurses were identified as having a moderate-high level of chronic stress more frequently because of dealing with patient loss several times a month (*φ* = 0.2, *p* = 0.019). In addition, as younger nurses tend to struggle more with mental health challenges, our study confirmed that the nurses with 0.5–9.0 years of work experience had a higher level of perceived stress compared to the subjects with 9.1–50 years of nursing experience (70.2% vs. 54.2%, *φ* = 0.2, *p* = 0.016).

### 3.2. Chronic Stress and Emotional States in Relation to Patient Death

The most competitive feelings and powerful emotions related to the death of a patient experienced by the nurses were compassion (73.1%), grief (60.1%), and helplessness (40.7%). In the interim, following the death of a patient, from 11 to 36% of the nurses’ emotions were associated with disappointment, despair, anger, guilt, anxiety, and calmness, while the smallest proportion, i.e., less than 6% of nurses, were indifferent or experienced a very depressed mood.

As displayed in Table 4, the logistic regression analyses disclosed the association between the emotions related to patient death and the perceived chronic stress levels in a cohort of nurses. After adjustment for the nursing experience (in years), the moderate–high level of the perceived chronic stress of nurses was associated with higher odds of emotions related to the death of a patient, namely, helplessness (AOR 1.7, 95% CI 1.1; 2.9), disappointment (AOR 1.9, 95% CI 1.1; 3.5), anxiety (AOR 1.9, 95% CI 1.2; 4.2), and guilt (AOR 4.7, 95% CI 1.4; 5.7). Conversely, the assessment of the sample of female nurses showed that the AOR for the lower level of chronic stress was related to the emotional reaction to the loss of a patient, specifically, calmness (AOR 0.6, 95% CI 0.3; 0.9).

**Figure A1:**
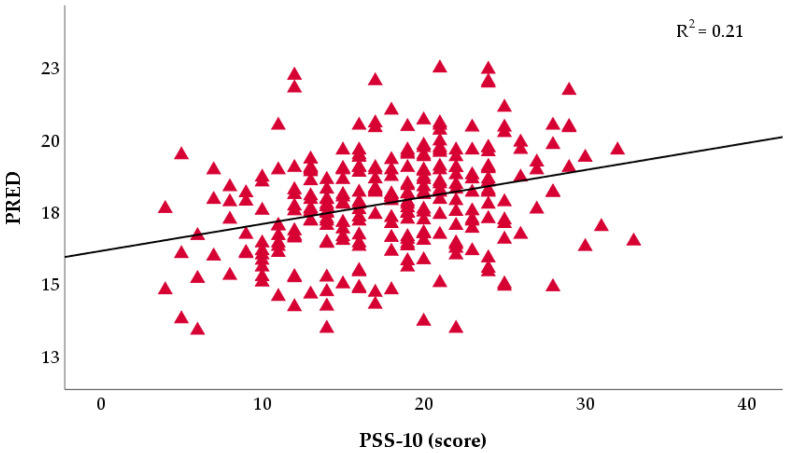
Graphic representation of multiple linear regression model (independent variable: average scores for expression of emotional complexity (PREDs)) of chronic stress; dependent variables are the PSS-10 scores. The multiple regression models were adjusted for nursing experience (in years). Model: F_2,282_ = 14.5, *p* < 0.001, *R*^2^ = 0.21, VIF = 1.0. PRED—unstandardized predicted value, F—the F-statistic, *R*^2^—the *R*-Squared, VIF—the variance inflation factor.

Figure A1 shows the linear regression analysis that identified the significant predicted values (PREDs) of complex emotions for the moderate–high chronic stress perceived by nurses (β 9.3, 95% CI 5.0; 13.6, *R*^2^ = 0.21).

### 3.3. Chronic Stress and Coping Styles 

**Figure 2:**
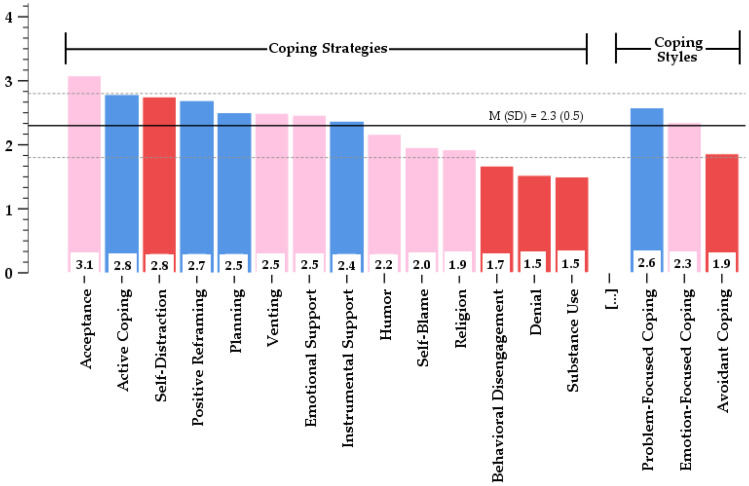
Mean Brief-COPE strategy scores in opposition to the mean Brief-COPE score of all items (M (SD)). Right 3 columns—coping styles; left 14 columns—coping strategies. Color codes: pink—‘emotion-focused’ coping; blue—‘problem-focused’ coping; red—‘avoidant’ coping.

Figure 2 shows the scores for three coping styles and fourteen coping strategies, ordered depending on to their scores. The total mean of all the Brief-COPE strategies was estimated to be 2.3 with a standard deviation of 0.5. Regarding nurses’ attitude to coping styles, the most prevailing styles consisted of problem-focused coping and emotion-focused coping with mean scores on the Brief-COPE of 2.6 (SD = 0.6) and 2.3 (SD = 0.5), respectively. In contrast, the mean score (M (SD) = 1.9 (0.6)) of the avoidant coping style was lower than the mean scores derived from both problem-focused coping (M_∆P-FC–AC_ = 0.7, 95% CI 0.6; 0.8, *p* < 0.001) and emotion-focused coping subscales (M_∆E-FC–AC_ = 0.5, 95% CI 0.4; 0.6, *p* < 0.001).

A more detailed analysis of the study data showed that the mean score of eight coping strategies was higher the total mean (SD): ‘acceptance’, ‘active coping’, ‘self-distraction’, ‘positive reframing’, ‘venting’, ‘use of emotional support’, and ‘use of instrumental support’. All coping strategies with the lowest scores such as ‘behavioral disengagement’, ‘denial’, and ‘substance use’ matched the style of avoidant coping.

**Figure 3:**
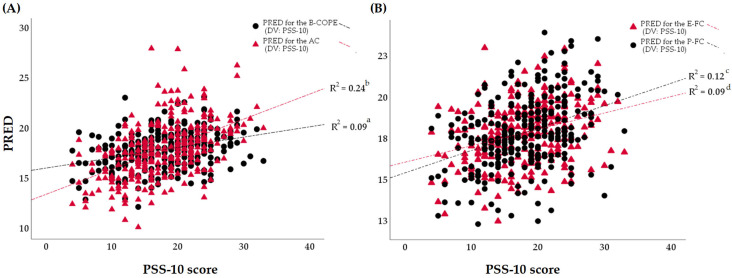
A graphic representation of multiple linear regression models (independent variables: different coping styles on chronic stress (PREDs); dependent variables are the PSS-10 scores. The multiple regression models were adjusted for nursing experience (in years), the rate for experiences with patient loss, and the expression of emotional complexity evoked in nurses by the death of a patient. (**A**): ^a^—Model (a): F_4,278_ = 7.9, *p* < 0.001, *R*^2^ = 0.09, VIF = 1.1; ^b^—Model (b): F_4,278_ = 23.2, *p* < 0.001, *R*^2^ = 0.24, VIF = 1.1; (**B**): ^c^—Model (c): F_4,278_ = 10.9, *p* < 0.001, *R*^2^ = 0.12, VIF = 1.0; ^d^—Model (d): F_4,278_ = 7.7, *p* < 0.001, *R*^2^ = 0.09, VIF = 1.1. See Table 3 for further details. PSS-10—the perceived stress scale, B-COPE—the brief-COPE scale, AC—the avoidant coping subscale, E-FC—the emotion-focused coping subscale, P-FC—the problem-focused coping subscale, PRED—the unstandardized predicted value, DV—the dependent variable, F—the F-statistic, *R*^2^—the *R*-Squared, VIF—the variance inflation factor.

Figure 3 displays multiple linear regression analyses that were represented according to the predicted values (PREDs) of the magnitude of self-perceived chronic stress resulting from a linear combination of the predictors, namely, different dimensions of the Brief-COPE scale. The confounding variables as covariates in the regression analysis were set as follows: nursing experience, dealing with the frequency of patient loss, and the expression of complex emotions following the death of a patient.

As shown in Table 5, although both the problem-focused coping (β −0.2, 95% CI −0.3; −0.1, *R*^2^ = 0.09) and the emotion-focused coping styles (β 0.1, 95% CI −0.1; −0.2, *R*^2^ = 0.12) were predominant, the *R*-squared values showed that the study data did not fit the regression model (in terms of *R*^2^ < 0.25 or < 25%). Therefore, the dimensions of emotion-focused coping and problem-focused coping were not associated with the lower levels of chronic stress in a sample of female nurses. Controversially, as indicated in Figure 3 and Table 5, the use of the avoidance coping style was associated with the moderate–high level of chronic stress perceived by nurses (β 0.5, 95% CI 0.4; 0.7, *R*^2^ = 0.24). In the avoidant coping subscale, a higher level of chronic stress perceived by nurses was only associated with the facet of ‘behavioral disengagement’ (β 3.0, 95% CI 2.3; 3.8, *R*^2^ = 0.26), which as a coping strategy that relies on ignoring or avoiding problematic situations or blocking out the emotions following perceived stress. Additionally, the results of this study identified a cognitive process, namely ‘self-blame’ (β 3.0, 95% CI 2.3; 3.7, *R*^2^ = 0.27), which was enrolled as the coping strategy applied by nurses more frequently following the occurrence of stressful events.

## 4. Discussion

### 4.1. Proportion of Chronic Stress in Clinical Nurses

In this study, 76% of the nurses had a moderate-to-high level of mental stress. Our study results matched the ones identified in Poland and India during the post-pandemic COVID-19 period when 76% to 81% of nursing staff reported a moderate-to-high level of perceived chronic stress, respectively [52,53]. Upon comparing the proportion of stressed nurses from Lithuania with the percentage (~43%) of caregivers suffering relevant mental stress symptoms from other 40 countries, the lower trends for the nurses’ self-reported perception of stress were observed during the COVID-19 pandemic [7]. The findings referring to the increased post-pandemic levels of chronic stress in caregivers also suggest a potential additional need for periodic screenings for perceived chronic stress in a cohort of nurses.

### 4.2. Emotional Experiences following Patient Death and Chronic Stress

Death along with the provision of end-of-life care to a patient during the dying process directly increase the acute stress perceived by nurses. The emotions practiced by caregivers serve as the basis for reflection as these experiences fall entirely into the nurses’ memory. The studies on emotional assessment are mostly carried out in a samples of nurses accompanying patients directly to the point of death (e.g., in oncology units and hospices) [54,55,56]. On the contrary, such types of research are very uncommon in terms of nurses working at other hospital departments [57,58]. According to our study results, the most powerful emotions such as compassion, grief, and helplessness were related to the death of a patient and experienced by the nurses working at other hospital departments (therapeutic, emergency, intensive care, and surgery units). By analogy, the results obtained in Polish nurses [58] on their emotional states, namely, compassion, sadness, and helplessness, after patient loss were almost identical compared to the psychological outcomes observed in Lithuanian nurses, who additionally felt more grief. Nevertheless, our study highlighted that not all the emotions experienced by nurses in the event of patient death were associated with the level of perceived chronic stress. Following patient death, the moderate–high level of the perceived chronic stress in nurses was related to emotional complexity as well as a rise in individual emotions such as helplessness, disappointment, anxiety, and guilt.

Although acute stress is not recognized as a risk factor for health, researchers have reached a consensus that, unlike positive emotions, negative emotions tend to accumulate faster [59] and have a relationship with mental acute stress [42]. Notwithstanding the evidence that chronic stress states take longer than emotional ones [40], the findings of our study can preliminarily extend this relationship according to the fact that negative emotions experienced by the nurses had links with chronic stress, too.

### 4.3. Styles and Strategies for Coping with Chronic Stress

Given that stress is defined as a persistent and unresolved ‘imbalance between stimulating and tranquilizing biochemicals’ [60], the condition of long-term stress may damage brain cells [23,61]. For this reason, coping strategies are necessary for nurses to handle both acute and chronic stress. It should be highlighted that the most prevailing styles included both problem-focused and emotion-focused coping; however, the overall use of these coping styles did not hold in relation to the level of exposure to a chronic stress in Lithuanian nurses we had studied.

According to our research, the most common active stress-coping strategies used by nurses were ‘acceptance’, ‘active coping’, ‘self distraction’, ‘positive reframing’, ‘venting’, ‘use of emotional support’, and ‘use of instrumental support’; at the same time, these ones were no different from the most commonly practiced coping strategies applied by nurses from other countries, namely Poland [62,63], Belarus [62], Australia [64], New Zealand [64], Southwest Ethiopia [65], and China [66]. In addition, it should be highlighted that Lithuanian clinical nurses had sufficient resources and developed problem-focused coping strategies; however, those active coping methods were not correlated to lower levels of perceived chronic stress. This means that, in practice, nurses should additionally adopt effective interventions related to art therapy, relaxation techniques, and Emotional Freedom Techniques (ECTs) as these ones may result in a positive effect on the levels of burnout [67].

Other scholars [62,63,64,65,66] have also found the avoidance coping style to be less used by nurses, but there are almost no reports [36] on the association between the use of maladaptive strategies and psychological or physiological outcomes in nurses. Controversially, according to our research, the less-used avoidant coping style had an inverse association between this type of maladaptive approach and the elevated chronic stress perceived by Lithuanian nurses. Furthermore, in the avoidant coping subscale, only one sub-dimension referring to the ‘behavioral disengagement’ strategy was associated with moderate-to-high levels of mental stress perceived by nurses.

The American Psychological Association (APA) defines disengagement as ‘the act of withdrawing from an attachment or relationship or, more generally, from an unpleasant situation’ [68]. While behavioral disengagement is a depressive symptom, it does not address the main problem in the development of chronic stress and may have dire impacts on mental health in the long term.

Finally, the nurses we studied tended to apply a ‘self-blame’ coping strategy, which, as part of the emotional-coping style, was related to severe symptoms of mental stress. The self-blame approach is a common reaction to stressful events and shows how nurses control perceived mental stress [69]. It has been hypothesized that self-blame activates other types of adaptive coping and has a positive correlation with maladaptive stress coping strategies [70]. Therefore, it is difficult to suppose that the encouragement of nurses’ self-blame may act as an effective stress coping strategy. On the contrary, the preventive measure related to self-blame ‘switching’ from personal factors to behavioral factors could benefit caregivers dealing with the death of a patient.

In terms of emotion-focused stress control, Lithuanian nurses have low control of perceived chronic stress, which indicates that the use of problem-focused coping has not been addressed to an appropriate extent either. Therefore, in order to promote mental health sustainability through additional social support interventions, it would be useful if the clinical nurses’ daily routines included therapies of mindfulness [71] that teach them ‘to let feelings and thoughts arise and let them go’.

### 4.4. Limitations and Future Research

Our study had several limitations. The first limitation of this study is associated with the fact that the causal relationship between perceived stress and potential risk factors related to both the use of coping strategies and the nurses’ emotional experiences after dealing with patient death should be assessed with caution as this study was cross-sectional in design. The second limitation of this study is related to the relatively small, although representative, sample size studied. In addition, the studied group came from three selected large cities in Lithuania, which makes it impossible to extrapolate the study results for larger cohorts of Lithuanian nurses. Although the *R²* value of 0.24 obtained from the primary multiple regression model indicated only a moderate fit, the likelihood of generalization of our study results should be assessed with caution. In addition, whilst the scientific literature recommends to categorize emotions based on their valence and arousal levels [45], our study included dichotomous questions for the assessment of emotional experiences of respondents. In line with this limitation, further research on the exploration of subjects’ true feelings and emotions could be carried out using the Self-Assessment Manikin (SAM) questionnaire [23,72]. Regardless of these limitations, the results of this study can serve as a starting point for further longitudinal or experimental studies on the effectiveness of social support interventions in managing both acute and chronic stress perceived by caregivers.

## 5. Conclusions

The study identified that more than half of the nurses experienced patient loss several times a month, were exposed to the perceived chronic stress, and suffered a moderate-to-high level symptoms of mental stress. The nurses’ emotional reactions to patient death were related to higher levels of the perceived chronic stress. Furthermore, psychological arousal following the death of a patient may induce the occurrence of emotional complexity that refers to competitive feelings, namely, helplessness, disappointment, anxiety, and guilt, and can serve as a potential trigger for the development of chronic stress in clinical nurses.

Regardless of the predomination of the nurses with a problem-focused coping profile in relieving perceived stress, the active copers did not have better psychological outcomes than the avoidant copers, implying that ‘doing something to cope with the stressor was better than doing nothing’. The caregivers were willing to use the emotion-focused coping style, however, without a better psychological outcome. In addition, the sub-dimension of the emotion-focused coping scale referred to a cognitive process of self-blame which, according to our study results, was considered a meaningful maladaptive behavior component magnifying chronic stress in nurses. Finally, after the differences in the use of a coping style were found, depending on the nurses’ self-reported perception of stress, the avoidant coping strategy related to behavioral disengagement was significantly associated with resilience to chronic stress in an adverse way. Therefore, our study highlights the need to implement recommendations for boosting nurses’ mental health. Hospitals should contribute to social support and psychological assistance along with training provided on stress-coping strategies for clinical nurses.

## Figures and Tables

**Table 2:** Levels of chronic stress perceived by the nurses.

PSS-10 Points	Sten	Perceived Stress Level	n	%
Below 7	1	Low	11	3.9
8–10	2		19	6.7
11–13	3		37	13.1
14–17	4	Moderate	66	23.3
18–20	5		57	20.1
21–23	6		43	15.2
24–25	7		28	9.9
26–27	8	High	7	2.5
28–30	9		12	4.2
Over 31	10		3	1.1

PSS—perceived stress scale.

**Table 3:** Categorization of nurses with different levels of self-perceived chronic stress by demographic and occupational characteristics and experiences with patient loss.

Variables	Self-Perceived Chronic Stress Levels				*φ* ^a^/*V* ^b^	*p*
	Low (Score: 0–13)		Moderate–High (Score: 14–40)			
	n	%	n	%		
Age (yr.) (M (SD))	40.9 (11.9)		36.1 (11.4)			
Age category						
20 to 37 years old	29	19.6	119	80.4	–0.1 ^a^	0.091
38 to 70 years old	38	28.1	97	71.9		
Educational attainment						
University	31	20.7	119	79.3	–0.1 ^a^	0.206
College	36	27.1	97	72.9		
Marital status						
In a relationship	15	22.4	52	77.6	0.1 ^b^	0.494
Divorced	4	22.2	14	77.8		
Married	31	21.1	116	78.9		
Widowed	2	40.0	3	60.0		
Single	15	32.6	31	67.4		
Workplace						
Intensive care unit	19	28.8	47	71.2	–0.1 ^b^	0.464
Surgical profile unit	7	28.0	18	72.0		
Therapeutic profile unit	17	25.0	51	75.8		
Emergency profile unit	24	19.4	100	80.6		
Nursing shifts						
Day shifts	14	21.9	50	78.1	–0.1 ^b^	0.683
Night shifts	8	19.5	33	80.5		
Mixture of day and night shifts	45	25.3	133	74.7		
Experiences with patient loss						
One time or several times a year	35	31.0	78	69.0	0.2 ^a^	0.019
Several times a month	32	18.8	138	81.2		
Nursing experience						
9.1–50 years	25	37.3	117	54.2	0.2 ^a^	0.016
0.5–9.0 years	42	29.8	99	70.2		

^a^—the Phi coefficient (*φ*); ^b^—the Cramer’s V correlation coefficient (*V*); *p*—*p*-value; M—mean, SD—standard deviation.

**Table 4:** Logistic regression analyses for feelings and emotions related to patient death (independent variables) with different levels of chronic stress (dependent variable) in clinical nurses.

Model	Independent Variable	β (SE)	Wald	*p*	AOR 95% CI (LB; UB)
1. PSS-10 (score: 14–40) ^a × 1^	Guilt (+) ^1^	1.6 (0.6)	6.3	0.012	4.7 [1.4; 5.7]
2. PSS-10 (score: 14–40) ^a × 2^	Compassion (+) ^2^	–0.2 (0.3)	0.4	0.530	0.8 [0.4; 1.5]
3. PSS-10 (score: 14–40) ^a × 3^	Disappointment (+) ^3^	0.6 (0.3)	5.2	0.039	1.9 [1.1; 3.5]
4. PSS-10 (score: 14–40) ^a × 4^	Sadness (+) ^4^	0.3 (0.3)	1.4	0.236	1.4 [0.8; 2.5]
5. PSS-10 (score: 14–40) ^a × 5^	Depressed mood (+) ^5^	1.7 (1.0)	2.6	0.110	5.3 [0.7; 8.6]
6. PSS-10 (score: 14–40) ^a × 6^	Despair (+) ^6^	0.8 (0.6)	2.1	0.144	2.3 [0.8; 6.7]
7. PSS-10 (score: 14–40) ^a × 7^	Calmness (+) ^7^	–0.5 (0.2)	5.9	0.045	0.6 [0.3; 0.9]
8. PSS-10 (score: 14–40) ^a × 8^	Anger (+) ^8^	0.8 (0.6)	2.1	0.144	2.3 [0.8; 2.7]
9. PSS-10 (score: 14–40) ^a × 9^	Helplessness (+) ^9^	0.6 (0.2)	5.1	0.041	1.7 [1.1; 2.9]
10. PSS-10 (score: 14–40) ^a × 10^	Grief (+) ^10^	0.02 (0.3)	0.1	0.944	1.0 [0.6; 1.8]
11. PSS-10 (score: 14–40) ^a × 11^	Anxiety (+) ^11^	0.7 (0.3)	5.8	0.049	1.9 [1.2; 4.2]

^a^—dependent variable for logistic regression models. ^1–11^—independent variables (feelings and emotions related to patient death) for logistic regression models. SE—standard error; Wald—the Wald test; *p*—*p*-value; AOR—adjusted odds ratio (AOR = e^β^); 95% CI–95% confidence interval; LB—lower bound; UB—upper bound); (+)—positive value. Models _(1–11)_: reference category: low level of chronic stress (score: ≤13); PSS—perceived stress scale. The logistic regression models were adjusted for nursing experience (in years). If AOR > 1 and AOR ≠ 1, it suggests a positive association between the independent and dependent variables. If AOR < 1 and AOR ≠ 1, it implies a positive association between the independent and dependent variables.

**Table 5:** Association between different coping styles and self-perceived chronic stress as a dependent variable in a cohort of nurses (multiple regression analyses).

Model	Independent Variable	β	95% CI [LB; UB]	*p*	F_4,278_	VIF	*R* ^2^
1. PSS-10 ^a^	Brief-COPE scale	0.04	[−0.02; 0.1]	0.170	7.9	1.1	0.09
1.1. PSS-10 ^b^	Avoidant coping	0.5	[0.4; 0.7]	<0.001	23.2	1.1	0.24
1.1.1 PSS-10 ^b × 1^	Denial ^1^	1.6	[0.7; 2.4]	<0.001	11.0	1.0	0.14
1.1.2. PSS-10 ^b × 2^	Behavioral disengagement ^2^	3.0	[2.3; 3.8]	<0.001	24.3	1.0	0.26
1.1.3. PSS-10 ^b × 3^	Self-distraction ^3^	1.4	[0.7; 2.2]	<0.001	11.3	1.1	0.14
1.1.4. PSS-10 ^b × 4^	Substance use ^4^	1.9	[1.1; 2.7]	<0.001	13.7	1.1	0.17
1.2. PSS-10 ^c^	Emotion-focused coping	0.1	[−0.1; 0.2]	0.360	10.9	1.1	0.12
1.2.1. PSS-10 ^c × 1^	Use of emotional support ^1^	−1.1	[−1.8; −0.3]	0.006	9.6	1.0	0.12
1.2.2. PSS-10 ^c × 2^	Humor ^2^	0.7	[−0.04; −1.4]	0.063	8.4	1.0	0.11
1.2.3. PSS-10 ^c × 3^	Religion ^3^	−0.2	[−0.8; 0.5]	0.646	7.5	1.0	0.09
1.2.4. PSS-10 ^c × 4^	Venting ^4^	−0.2	[−0.9; 0.6]	0.669	7.5	1.0	0.09
1.2.5. PSS-10 ^c × 5^	Acceptance ^5^	−1.2	[−2.1; −0.4]	0.006	9.5	1.0	0.12
1.2.6. PSS-10 ^c × 6^	Self-blame ^6^	3.0	[2.3; 3.7]	<0.001	26.8	1.2	0.27
1.3. PSS-10 ^d^	Problem-focused coping	−0.2	[−0.3; −0.1]	<0.001	7.7	1.0	0.09
1.3.1. PSS-10 ^d × 1^	Active coping ^1^	−1.1	[−2.1; −0.1]	0.027	6.7	1.0	0.08
1.3.2. PSS-10 ^d × 2^	Positive reframing ^2^	−1.4	[−2.2; −0.6]	0.001	10.8	1.0	0.14
1.3.3. PSS-10 ^d × 3^	Use of instrumental support ^3^	−0.5	[−1.2; −0.3]	0.198	7.9	1.0	0.10
1.3.4. PSS-10 ^d × 4^	Planning ^4^	−0.2	[−0.9; 0.6]	0.645	7.5	1.0	0.10

^a–d^—dependent variables for multiple linear regression models. ^1–6^—independent variables (in terms of 3 styles and 14 strategies of Brief-COPE) for multiple linear regression models. All regression models were adjusted for nursing experience (in years), the rate of experiences with patient loss, and the expression of emotional complexity evoked in nurses by the death of a patient. PSS-10—the perceived stress scale, *p*—*p*-value, 95% CI—95% confidence interval, LB—lower bound, UB—upper bound, F—the F-statistic, *R*^2^—the *R*-Squared, VIF—the variance inflation factor.

## Data Availability

Data are available on request.

## Nomenclature

**Term**	**Definition**
AC	Avoidant Coping Subscale
APA	American Psychological Association
B-COPE	Brief-COPE Scale
CI	Confidence Interval
COPE	Coping Orientation to Problems Experienced
COVID-19	Coronavirus Disease
DSM-5	Diagnostic and Statistical Manual of Mental Disorders
DV	Dependent Variable
ECT	Emotional Freedom Techniques
E-FC	Emotion-Focused Coping Subscale
yr.	Year
F	F-Statistic
LB	Lower Bound
*P*	*p*-value
*ɸ*	Phi Coefficient
M	Mean
mo.	Month
AOR	Adjusted Odds Ratio
P-FC	Problem-Focused Coping Subscale
PRED	Unstandardized Predicted Value
PSS-10	Perceived Stress Scale
*R* ^2^	*R*-Squared
SAM	Self-Assessment Manikin
SD	Standard Deviation
SPSS	Statistical Package for the Social Sciences
STROBE	Strengthening the Reporting of Observational Studies in Epidemiology
UB	Upper bound
*V*	Cramer’s V Correlation Coefficient
VIF	Variance Inflation Factor
vs.	Versus
WHO	World Health Organization
